# Equine Gastric Ulcer Syndrome affects fitness parameters in poorly performing Standardbred racehorses

**DOI:** 10.3389/fvets.2022.1014619

**Published:** 2022-11-25

**Authors:** Chiara Maria Lo Feudo, Luca Stucchi, Bianca Conturba, Giovanni Stancari, Enrica Zucca, Francesco Ferrucci

**Affiliations:** ^1^Equine Sports Medicine Laboratory “Franco Tradati”, Department of Veterinary Medicine and Animal Sciences, Università Degli Studi di Milano, Lodi, Italy; ^2^Veterinary Teaching Hospital, Department of Veterinary Medicine and Animal Sciences, Università Degli Studi di Milano, Lodi, Italy

**Keywords:** EGUS, poor performance, fitness capacity, equine sports medicine, gastric ulcer, treadmill test, racehorses

## Abstract

**Introduction:**

Equine Gastric Ulcer Syndrome (EGUS) is a highly prevalent disorder in horses, which can be classified, based on the localization of the lesions, as Equine Squamous Gastric Disease (ESGD) or Equine Glandular Gastric Disease (EGGD). Although EGUS is recognized as a common cause of poor performance in racehorses, objective investigations about its relation with athletic capacity are lacking. Therefore, the present retrospective study aims to evaluate the associations between EGUS severity and some fitness parameters measured during an incremental treadmill test in Standardbred racehorses in training.

**Methods:**

With this aim, data from 87 Standard bred racehorses which underwent a complete diagnostic evaluation for poor performance was reviewed. During gastroscopic examination, a 0-4 score was assigned to ESGD, while EGGD was evaluated for absence/presence; a total EGUS score was obtained by adding 1 point to ESGD score in horses showing concomitant EGGD. Fitness parameters obtained during incremental treadmill test included speed at a heart rate of 200 bpm (V200), speed and heart rate at a blood lactate of 4 mmol/L (VLa4, HRLa4), peak lactate, lactate and heart rate at 30 minutes post-exercise, maximum speed, minimum pH and maximum hematocrit. The associations between fitness parameters and EGUS and ESGD scores were evaluated by Spearman correlation, while Mann-Whitney test was used to compare them between horses with or without EGGD. Statistical significance was set at *p*<0.05.

**Results:**

EGUS grade was inversely correlated with V200 (*p* = 0.0025) and minimum pH (*p* = 0.0469); ESGD grade was inversely correlated with V200 (*p* = 0.0025) and VLa4 (*p* = 0.0363). Although a trend was observed, no significant differences in V200 were observed between horses with or without EGGD (*p* = 0.073); horses with EGGD reached a lower minimum pH (*p* = 0.0087).

**Discussion:**

These results show a negative association between aerobic capacity and EGUS, in particular ESGD. Although different hypotheses have been proposed, including abdominal pain and decreased appetite due to lactate accumulation, the underlying mechanisms are still unknown, and it is not clear whether EGUS represents a cause or a consequence of an early lactate accumulation and post-exercise acidosis.

## Introduction

Equine Gastric Ulcer Syndrome (EGUS) is a common disease in horses, defined as the presence of erosive and ulcerative lesions in the terminal esophagus, proximal squamous stomach, distal glandular stomach, and proximal duodenum (1, 2). Based on the affected anatomical region, two different forms of EGUS have been distinguished: Equine Squamous Gastric Disease (ESGD) and Equine Glandular Gastric Disease (EGGD), which differ in their pathophysiological mechanisms (1). The prevalence of EGUS varies depending on breed, attitude, training, and localization of the lesions, with the squamous mucosa being the most commonly affected site (3–7); racehorses are the most represented breeds (8), reaching a prevalence of up to 95% during intense training and racing periods. As a matter of fact, exercise frequency is one of the main risk factors for EGUS development (4, 6, 9–15). An increased risk of EGGD has been associated with the number of days exercised per week (10, 16), as well as with the level of events in which horses compete (16); similarly, ESGD development is predisposed by higher frequency (6, 10, 16), duration (10), and intensity (6) of exercise. During intensive physical activity, the increased intra-abdominal pressure causes gastric compression, pushing the gastric content cranially and exposing the squamous mucosa to acids, which may lead to its ulceration (1, 6, 10, 12–15, 17). Moreover, intraluminal pH decreases in the proximal portion of the stomach as exercise intensity increases, leading to higher odds of developing ESGD (6, 18), and serum gastrin concentration has been shown to increase during exercise, stimulating hydrochloric acid secretion (19). A higher risk of EGGD has been reported in Thoroughbred racehorses exercising 5–7 days per week, compared to those exercising 1–4 days per week (10), and decreasing exercise duration and frequency has been suggested as a management tool for EGGD prevention (11). It has been hypothesized that the association between training and EGGD may be due to the stress associated with intensive exercise (11, 13), as sport horses with EGGD show a higher response to ACTH stimulation (20); another possible explanation is that, as exercise intensity increases, blood flow shifts away from the gastric mucosa to skeletal muscles, determining a reduced gastric motility and perfusion (21).

Clinical signs of EGUS are usually subtle and non-specific (22–28). Among them, the possible role of EGUS as a cause of poor performance in racehorses is of particular interest (23, 25, 29–31): although widely recognized among researchers, veterinarians, and trainers, to date few studies have evaluated the potential effects of the presence and severity of EGUS on sport performance (1). In a study, EGUS was identified as the only cause of poor performance in four racehorses, and treatment with omeprazole was effective in improving performance (30). In particular, these horses showed concomitant ESGD and EGGD of different severity and were referred for decreased willingness to gallop or recent history of slowing or stopping suddenly toward the end of races; after medical treatment and management modifications, the horses were reported to be more willing to work, and either were placed or won the following races. A similar improvement of performance after omeprazole administration, defined as better placements in races, was reported by other authors in a population of Thoroughbred racehorses (32). Other studies reported poor performance in Thoroughbred and Standardbred racehorses with EGUS, but performance evaluation only relied on trainers' expectations and was defined as above or below expectations (23, 29). In order to evaluate objectively and in a measurable way the effects of gastric ulceration on athletic performance, a study was conducted on Thoroughbred racehorses with experimentally induced ESGD, undergoing a standardized treadmill test; the results showed that ESGD was associated with decreased aerobic capacity, stride length and time to fatigue (33). Similar findings have been reported in human medicine: in fact, among human athletes, gastroesophageal reflux disease (GERD), which shares several similarities with ESGD (1, 23, 34–36), is very common and has been associated with decreased performance and time to exhaustion; however, the exact mechanisms by which GERD might affect performance have not been clarified yet (37).

In equine medicine, studies objectively investigating physiological responses to incremental treadmill exercise tests in horses with naturally occurring EGUS are lacking. Therefore, the present retrospective study aims to evaluate the potential impact of naturally occurring ESGD and EGGD on horses' athletic capacity, by measuring some fitness parameters during a standardized incremental treadmill test in poorly performing Standardbred racehorses.

## Materials and methods

### Horses

The clinical records of Standardbred racehorses referred for poor performance evaluation to the Equine Sports Medicine Unit of the Veterinary Teaching Hospital of the University of Milan (Italy) between 2002 and 2021 were retrospectively reviewed. As all the procedures were performed on clinical patients for diagnostic purposes and included informed owner consent for the use of clinical data, ethical review and approval were waived. All horses were in full training upon admission and underwent the following diagnostic protocol:

- Day 1: collection of history, clinical examination and lameness evaluation, laboratory analyses, electrocardiogram at rest, upper airway endoscopy at rest, acclimation on treadmill (one or two sessions);
- Day 2: incremental treadmill metabolic test, including plasma lactate analysis, creatine-kinase (CK) measurement at 6 h after exercise and Holter registration;
- Day 3: dynamic upper airway endoscopy on high-speed treadmill and tracheobronchoscopy performed 30 min after exercise for the evaluation of exercise-induced pulmonary hemorrhage (EIPH);
- Day 4: lower airway endoscopy, bronchoalveolar lavage fluid (BALf) collection and cytological examination;
- Day 5: gastroscopic examination.

Horses showing signs of systemic illness, lameness, clinically significant cardiac arrhythmias or valvular regurgitation, dynamic upper airway obstructions (DUAO) or rhabdomyolysis were excluded from the study, since these disorders may influence athletic performance. As lower airway inflammation is highly prevalent among racehorses, its presence was not an exclusion criterium: however, BALf cytological findings were taken into consideration during statistical analysis as a possible bias.

### Incremental treadmill metabolic test

On Day 1 the horses were conditioned to the high-speed treadmill (Sato I, Uppsala, Sweden) with one or two training sessions. On Day 2, the incremental treadmill metabolic test was performed: the belt was inclined with a 3° slope and horses were warmed-up with 4-min walk at 1.5 m/s and 3-min trot at 6 m/s. The warm-up was followed by 1-min phases, during which the speed was increased by 1 m/s, until the horse was no longer able to maintain the treadmill speed; at the end of the test, the horses were walked for 30 min with a 0° slope to cool down (38).

During the treadmill test, blood samples were collected with the aid of a 14 G Teflon venous catheter placed in the left jugular vein and connected to an extension tube: blood samples were taken at rest, after the warm-up phase, at the end of each speed phase, and at 1, 5, 15, and 30 min during the cool down. To perform plasma lactate analysis, blood was transferred into tubes containing 10 mg sodium fluoride and 2 mg potassium oxalate for 1 mL of blood; samples were centrifugated within 15 min and refrigerated, and plasma lactate was measured with the enzymatic colorimetric method, using a lactate dry-fast kit for the automatic system (Uni Fast System II Analyzer, Sclavo, Italy) and reagents supplied by the manufacturer (39). In some horses, an aliquot of blood collected in heparinized syringes at each phase was used to measure blood pH (*n* = 56) and hematocrit (*n* = 44), by means of a blood gas analyzer (Opti CCA, Opti Medical System, Roswell, USA) (38).

Throughout the duration of the treadmill test, the heart rate was monitored in real-time using a heart rate monitor (Polar, Equine Inzone FT1, Steinhausen, Switzerland); moreover, an ECG was obtained continuously before, during and after exercise, by means of a Holter recorder (Cardioline^®^ Click Holter, Trento, Italy) (40). To exclude the presence of rhabdomyolysis, CK activity was measured 6 h after the end of the test; a blood sample was collected, immediately centrifuged (PLC-02, Gemmy Industrial Corporation, Taipei, Taiwan), and serum was obtained. Serum CK activity was measured at 37°C with an automatic kinetic spectrophotometric UV method (Uni Fast System II Analyzer, Sclavo, Siena, Italy), using reagents supplied by the manufacturer; CK values between 44 and 735 U/L were considered within normal limits (41).

The fitness parameters obtained from the treadmill test were collected on an electronic sheet (Microsoft Excel, Redmond, USA), and included:

- VLa4: speed at a plasma lactate concentration of 4 mmol/L;
- HRLa4: heart rate at a plasma lactate concentration of 4 mmol/L;
- V200: speed at a heart rate of 200 bpm;
- Lac 30: plasma lactate concentrations at 30 min during the cool down;
- HR 30: heart rate at 30 min during the cool down;
- Lac max: maximum plasma lactate concentration reached during treadmill test or cool down;
- V max: maximum speed reached during the test until fatigue;
- pH min: minimum pH reached during the test;
- Ht max: maximum hematocrit reached during the test.

The values of VLa4 and HRLa4 were calculated by means of a specific software (Lactate-E 1.0), which provides precise lactate threshold markers by inverse prediction (42).

### Airway endoscopy, BALf collection and cytological examination

A complete evaluation of the upper and lower airways was included as part of the diagnostic protocol for poor performance. On Day 1, the anatomy and function of the upper airways were evaluated through an endoscopic examination at rest: horses were restrained in stocks without pharmacological restraint, a flexible videoendoscope (EC-530WL-P, Fujifilm, Tokyo, Japan) was passed through the left nasal passage, and the upper tract of the respiratory tract was visualized. Laryngeal function was assessed during spontaneous breathing and after the stimulation of movements by inducing swallowing, performing nasal occlusion, and during the “slap test” (43). Horses showing persistent epiglottic entrapment or grade IV recurrent laryngeal neuropathy were excluded from the study.

On Day 3, a high-speed treadmill endoscopy (HSTE) was performed: after 4-min walk and 5-min trot warm up with a 5% slope, treadmill was temporarily stopped, a videoendoscope (ETM PVG-325, Storz, Tuttlingen, Germany) was passed into the nasopharynx of the horse and held in position with straps. Then, the treadmill was rapidly accelerated up to maximal speed until the horse's fatigue. The endoscopic images were visualized in real-time on a monitor and digitally recorded to allow slow-motion analysis (43). Horses showing any form of DUAO were excluded from the study. Thirty minutes after the end of the HSTE, horses underwent a tracheobronchoscopy: with this aim, they were restrained in stocks and with a twitch, endoscopy was performed as described above, the lower respiratory tract was examined and the possible presence of blood in the trachea was recorded (38).

On Day 4, a lower airway endoscopy was performed after sedation with detomidine hydrochloride (0.01 mg/kg IV), as previously described, and a BALf sample was collected for cytological examination: briefly, at the level of the carena, 60 mL of a 0.5% lidocaine hydrochloride solution were sprayed to inhibit the cough reflex, and the endoscope was passed into the bronchial tree until it was wedged within a segmental bronchus; here, 300 mL sterile saline 0.9% was instilled, and the fluid immediately aspirated. The collected BALf was stored in sterile EDTA tubes and processed within 90 min. A few drops of pooled BALf were cytocentrifugated (Rotofix 32, Hettich Cyto System, Germany) at 26 *g* for 5 min. The slides were air dried, stained with May-Grünwald Giemsa and Perl's Prussian blue, and observed under a light microscope at 400× and 1,000× for 400-cell leukocyte differential count and calculation of a simplified total hemosiderin score (38).

### Gastroscopic examination

Before gastroscopic examination, horses were starved for at least 8 h, while they had free access to water up to the time of examination (44, 45). To perform the gastroscopy, horses were contained in stocks, restrained with a twitch and sedated with detomidine hydrochloride (0.01 mg/kg IV). A videogastroscope (PV-G 34-325, Storz, Tuttlingen, Germany), connected to an aspirator pump (208-ACH, Faset, Trezzano sul Naviglio, Italy), was passed through the left nasal passages, nasopharynx, esophagus, until the stomach was visualized. To enable observation of the squamous and glandular mucosae, the *margo plicatus* and the pylorus, the stomach was insufflated with air and the mucosa was rinsed of adherent food material and mucus with water (46). The squamous mucosa was graded for ESGD using the Equine Gastric Ulcer Council 0–4 scoring system (2), while the glandular mucosa was evaluated for presence or absence of EGGD, as recommended by the European College of Equine Internal Medicine Consensus Statement (1). All scores were assigned by a single investigator (F.F.), who remained blinded to the incremental treadmill test results.

### Statistical analysis

All data were analyzed using descriptive statistics and evaluated for normality by means of the Shapiro-Wilk test. The influence of age and weight on fitness parameters, ESGD score, and BALf cytological findings was evaluated using the Spearman's correlation; moreover, age and weight were compared between EGGD-positive and EGGD-negative horses respectively with the Mann-Whitney test and the unpaired *t*-test. The influence of sex on fitness parameters, ESGD score, and BALf cytological results was evaluated by means of the Kruskal-Wallis test and Dunn's multiple comparisons test, while sex distribution was compared between EGGD-positive and EGGD-negative horses using the Chi-square test. The association between BALf leukocyte populations and fitness parameters was evaluated using the Spearman's correlation. The associations of ESGD grade with fitness parameters and BALf cytological results were evaluated using the Spearman's correlation. Based on the distribution of data, fitness parameters and BALf cytological results were compared between EGGD-positive and EGGD-negative horses by means of the Mann-Whitney test or the unpaired *t*-test. To assess the influence of mild-moderate equine asthma (MEA) on fitness parameters, horses were divided into MEA and non-MEA groups; horses were included in the MEA group when presenting a percentage of BALf neutrophils >10% and/or eosinophils >5%, and/or metachromatic cells >5% (47). Fitness parameters were compared between MEA and non-MEA groups by means of the unpaired *t*-test or the Mann-Whitney test, based on data distribution. Moreover, the possible association between MEA and EGUS was evaluated by comparing ESGD scores between groups using the Mann-Whitney test and comparing the frequency of EGGD between groups by means of the Fisher's exact test.

Data are presented as mean ± standard deviation (SD) if normally distributed and as median and interquartile range (IQR) if not normally distributed. Statistical significance was set at *p* < 0.05. Data were analyzed using a commercially available statistical software package (GraphPad Prism 9.1.0 for MacOS; GraphPad Software, San Diego, CA, USA).

## Results

### Study population

Eighty-seven Standardbred racehorses met the inclusion criteria. The study population consisted of 10 geldings, 43 stallions and 34 mares, aged from 2 to 8 years (median 3 years, IQR 3–4 years), and weighing from 373 to 527 kg (mean 445.7 ± 32.39 kg). The fitness parameters obtained by the incremental treadmill metabolic test and the results of BALf cytology are displayed in Tables 1, 2 respectively. MEA was diagnosed in 72.4% of the horses, while BALf cytology was within normal limits in 27.6%.

**Table 1:** Results of fitness parameters obtained during the incremental treadmill metabolic test in our study population.

**Fitness parameter**	**Value**
V200 (m/s)	8 (7–8.5)
VLa4 (m/s)	8.3 (7.1–9)
HRLa4 (bpm)	205.7 (194.8–213.7)
Lac 30 (mmol/L)	11.55 (6.97–16.11)
HR 30 (bpm)	73.8 ± 18.65
Lac max (mmol/L)	21.17 ± 7.68
V max (m/s)	(11–11)
pH min	7.16 ± 0.1
Ht max (%)	64.5 (61-68)

Data are expressed as mean ± standard deviation if normally distributed and as median (interquartile range) if not normally distributed.

**Table 2:** Results of BALf cytological examination in our study population.

**BALf leukocyte count**	**Value**
Macrophages (%)	45.3 ± 8.55
Lymphocytes (%)	34.54 ± 11.55
Neutrophils (%)	13 (7–20)
Eosinophils (%)	1 (0–2.75)
Mast cells (%)	4 (3–5)
Total hemosiderin score	38(14-78)

Data are expressed as mean ± standard deviation if normally distributed and as median (interquartile range) if not normally distributed.

### Gastroscopy

The squamous and glandular mucosae, the *margo plicatus*, and the pyloric antrum were visualized and evaluated in all horses. In the study population, ESGD had a prevalence of 98.85% (95% CI: 93.77–99.94%). In particular, no horses showed grade 1 ESGD, 8.05% of horses showed grade 2 ESGD, 21.84% grade 3, and 68.96% grade 4 (median 4, IQR 3–4). The presence of EGGD was observed in 46 horses (52.87%; 95% CI: 42.49–63.02%), while no lesions of the glandular mucosa were detected in 41 horses (47.13%). All horses with EGGD were concomitantly affected by ESGD.

### Influence of age, sex, and weight

No association between ESGD grade or presence/absence of EGGD and age, sex, nor weight was observed. Age was positively correlated with the values of VLa4 (*p* = 0.0457, *r* = 0.21), V max (*p* = 0.0014, *r* = 0.34) and Ht max (*p* = 0.0149, *r* = 0.36); moreover, it was inversely correlated with BALf eosinophils count (*p* = 0.0123, *r* = −0.29). Sex and weight were not associated with any fitness parameters, or BALf cytology results.

### Influence of BALf cytology

No relationship was detected between BALf cytological results and ESGD grade, or presence/absence of EGGD. Moreover, no association was observed between presence/absence of MEA and ESGD grade, or presence/absence of EGGD. The count of BALf macrophages was inversely correlated with the values of VLa4 (*p* = 0.0304, *r* = −0.25), HRLa4 (*p* = 0.0004, *r* = −0.41), and Ht max (*p* = 0.0346, *r* = −0.36). The BALf lymphocyte percentage was positively correlated with the values of VLa4 (*p* = 0.001, *r* = 0.38), HRLa4 (*p* = 0.0002, *r* = 0.43), and V max (*p* = 0.0205, *r* = 0.27); conversely, it was inversely correlated with the value of Lac 30 (*p* = 0.0055, *r* = −0.33). The BALf neutrophil count was inversely correlated with the values of VLa4 (*p* = 0.0037, *r* = −0.34), HRLa4 (*p* = 0.0365, *r* = −0.25), V max (*p* = 0.0155, *r* = −0.29), and positively correlated with Lac 30 (*p* = 0.0024, r = 0.36) and Lac max (*p* = 0.0084, *r* = 0.31). The percentages of BALf eosinophils and mast cells and the total hemosiderin score were not associated with any fitness parameter. When evaluating MEA and non-MEA groups, horses in the MEA group showed lower values of VLa4 (*p* = 0.0341), while no other fitness parameter differed between groups.

### Gastroscopy vs. fitness parameters

The ESGD grade was inversely correlated with V200 (*p* = 0.0016, *r* = −0.33) and VLa4 (*p* = 0.0250, *r* = −0.24). The presence of EGGD was associated with lower values of pH min (*p* = 0.012).

## Discussion

Equine Gastric Ulcer Syndrome is widely recognized as a common cause of poor performance in racehorses; however, to date, no studies have objectively investigated the association between athletic capacity and the natural occurrence of EGUS. Therefore, to the authors' knowledge, the present study is the first reporting the relationship existing between several fitness parameters and the presence and severity of EGUS in Standardbred racehorses.

Among racehorses in active training, reported ESGD prevalence varies from 70 to 100% (4, 5, 10, 23, 29, 48), while EGGD has been reported in 25–65% of racehorses (10, 48, 49); in general, due to the differences in the pathogenetic mechanisms, the squamous mucosa adjacent to the *margo plicatus* is the most commonly affected site (6, 21, 23). In our study, ESGD showed a prevalence of 98.85%, while EGGD affected 52.87% of the horses: the prevalence of both forms of EGUS was similar to some previous reports, but slightly higher compared to others. This can be explained by the fact that all horses enrolled in the present study were poorly performing, therefore they were more likely to suffer from EGUS (10); conversely, previous studies were performed on populations including both highly and poorly performing racehorses. Moreover, horses concomitantly affected by other disorders potentially impacting on performance were excluded from the present study, except for those affected by MEA; therefore, the probability that the cause of poor performance was EGUS was higher than in an unbiased racehorse population. It is thus reasonable that the inclusion criteria of the present study may have influenced the results concerning ESGD and EGGD prevalence, which should be interpreted accordingly; however, the aim of the present study was not to report the epidemiology of these diseases.

The high prevalence of EGUS makes it a very common cause of decreased athletic performance among racehorses (11, 23, 25, 29–31); although this relationship is largely accepted among the equestrian and veterinary communities, previous studies mainly relied on the trainers' expectations or racing placements for performance evaluation (23, 29, 30, 32), and the only study objectively measuring fitness parameters was performed on horses with experimentally induced ESGD (33). The present study is the first to demonstrate an association between severity of naturally occurring EGUS and decreased fitness in Standardbred racehorses. In particular, the values of VLa4 and V200 were lower in horses with higher ESGD grades, suggesting an impairment in aerobic capacity; similarly, Nieto et al. (33) reported a higher maximum oxygen consumption, which is considered the best parameter for aerobic capacity, in ulcer-free horses compared to horses with experimentally induced ESGD, during a standardized treadmill test. Moreover, in our study, EGGD was associated with a lower minimum pH during the treadmill test, and a trend was noticed for a higher peak plasma lactate concentration. These findings suggest that, in horses with EGGD, the anaerobic metabolism may be used longer during exercise, compared to non-affected horses, determining a prolonged lactate accumulation and, therefore, a more severe blood acidosis. In the study by Nieto et al. (33), a higher plasma lactate accumulation rate and a shorter time to fatigue were observed in horses with gastric ulceration compared to ulcer-free horses, although statistical significance was not reached; moreover, stride length, which has been reported as one of the strongest indicators of performance in racehorses, increased more in horses without ulceration in comparison with ESGD-affected horses after a treadmill exercise training program. These findings seem to confirm that EGUS, both when localized in the squamous mucosa and in the glandular mucosa, has a negative impact on fitness parameters, and in particular on aerobic capacity. The mechanisms by which EGUS affects performance have not been clarified yet; however, different hypotheses may be proposed. One possible explanation is that abdominal discomfort, reported as a common clinical sign of EGUS (23, 24, 26–28), may prevent affected horses from making their best effort in training or racing (as suggested by the shorter stride length). Moreover, abdominal discomfort or pain may also affect diaphragmatic excursion during breathing, decreasing tidal volume and alveolar ventilation: if blood gas exchanges at the alveolar level is impaired, aerobic capacity would be affected and an earlier switch to anaerobic metabolism may occur. This mechanism may explain the lower values of VLa4 and V200 in horses with ESGD, and the higher peak lactate in horses with EGGD, detected in our study. It has also been hypothesized that poor performance may be a consequence of the decreased food intake in EGUS-affected horses (33): in fact, lack of appetite is very common among horses with gastric ulceration, and an inappropriate energy intake may be detrimental for athletic performance. Interestingly, recent studies in human medicine demonstrated that post-exercise lactate accumulation may mediate appetite suppression and subsequent reduced energy intake (49): in horses with EGGD, a greater lactate production may contribute to post-exercise inappetence, subsequently leading to decreased performance and impaired mucosal healing. Finally, it is recognized that gastric ulcers develop in an acidic environment: it is possible that a lower gastric pH in EGUS-affected horses may lead to a different microbiome, as reported by a recent study (50); a diverse gastroenteric microbiome has recently proved to be critical for optimal fitness, therefore its alterations may impact negatively on athletic performance (51). Nevertheless, all these hypotheses do not elucidate how the specific localizations of gastric lesions affect different fitness parameters; unfortunately, the exact pathogenetic mechanisms occurring in EGGD are not fully understood, nor the differences in clinical signs associated with ESGD or EGGD. Therefore, possible theories regarding their different effects on fitness parameters would be highly speculative.

In human medicine, GERD is very common and affects up to 60% of elite athletes; affected athletes complain about upper gastroenteric pain, which increases proportionally to exercise intensity, a symptom known as *heart burn* (35, 52). This disease consists of reflux of stomach acids onto the sensitive squamous esophageal mucosa, which is histologically similar to the gastric squamous epithelial mucosa of horses; therefore, GERD is considered the human equivalent of ESGD (1, 23, 34–36). During intense exercise, in both human athletes and racehorses, abdominal pressure increases, pushing gastric contents proximally, exposing the squamous mucosa to acids (35). Similarly to what happens in horses with gastric ulcers, GERD has been associated with poor performance in human runners, and in particular with a decreased time to exhaustion (37). Among human athletes, decreased performance seems to be a consequence of esophageal and chest pain during exercise (23, 35). Some authors investigated the possible role of increased airway resistance, associated with GERD, in decreased performance, but an experimental study refuted this hypothesis (35). The reason underlying this idea is that GERD has long been associated with asthma (53–56); hypotheses have been formulated to explain this relationship, including bronchoconstriction induced by microaspiration of gastric acids by reflux (57–59), or neurogenic activation of bronchospasm due to acid stimulation of esophageal nerves (60–62). However, in our study no association was observed between EGUS and neutrophilic, eosinophilic, mastocytic or mixed lower airway inflammation; therefore, in equine medicine, the role of EGUS in poor performance does not seem to be associated with asthma-related bronchoconstriction. The inclusion of asthmatic horses in the present work is probably the main limitation of the study: as a matter of fact, BALf neutrophilia and mastocytosis have been previously shown to negatively affect performance (63), and neutrophilia has also been associated with a lower VLa4 (39). However, lower airway inflammation has a prevalence of up to 80% among racehorses (63), and it would not have been possible to exclude affected horses without reducing dramatically the population size. In our study, MEA was associated with lower values of VLa4; this result shows an influence of lower airway inflammation on aerobic capacity, as previously reported (39, 63). However, in our study, no differences in the BALf differential cell count were observed between different grades of ESGD, nor between horses with or without EGGD; similarly, no differences in ESGD scores nor EGGD frequency were detected between MEA and non-MEA horses. Therefore, it could be hypothesized that the contribution of lower airway inflammation in impairing fitness parameters was equal in horses affected by different grades and localization of EGUS and did not affect the results of the study. Another limitation of the present study may be related to the fact that gastroscopy was performed at the end of a complete diagnostic protocol, including multiple exercise tests on treadmill. As gastric lesions may develop rapidly and their development and worsening have been associated with exercise frequency and intensity, it is possible that, in our study, EGUS may have been induced or worsened by treadmill exercise and so, it may have been over-diagnosed.

In conclusion, the present study confirms the negative impact of EGUS on performance in racehorses. However, the mechanisms by which ESGD and EGGD affect athletic capacity have not been elucidated yet, nor the possible differences between the modalities by which the two forms influence performance. Unfortunately, also in human medicine, only speculative hypotheses have been proposed regarding the mechanisms by which GERD, the human version of ESGD, affects performance. Further research should be undertaken to clarify the reasons why EGUS contributes to cause poor performance in racehorses.

## Data availability statement

The raw data supporting the conclusions of this article will be made available by the authors, without undue reservation.

## Ethics statement

Ethical review and approval was not required for the animal study because the present manuscript consists of a retrospective study and all the procedures were performed on clinical patients (horses) for diagnostic purposes, while no procedures were performed for research aims. Moreover, all the procedures were performed according to relevant guidelines as a part of standard diagnostic protocols. Written informed consent was obtained from the owners for the participation of their animals in this study.

## Author contributions

CL, LS, and FF contributed to conceptualization and design of the study. CL visualized, defined the methodology of the study, and performed formal analysis. CL, LS, BC, GS, EZ, and FF contributed to investigation. CL wrote the first draft of the manuscript, under the supervision of LS and FF. All authors contributed to manuscript revision, read, and approved the submitted version.

## Conflict of interest

The authors declare that the research was conducted in the absence of any commercial or financial relationships that could be construed as a potential conflict of interest.

## Publisher's note

All claims expressed in this article are solely those of the authors and do not necessarily represent those of their affiliated organizations, or those of the publisher, the editors and the reviewers. Any product that may be evaluated in this article, or claim that may be made by its manufacturer, is not guaranteed or endorsed by the publisher.
